# A new resolution function to evaluate tree shape statistics

**DOI:** 10.1371/journal.pone.0224197

**Published:** 2019-11-21

**Authors:** Maryam Hayati, Bita Shadgar, Leonid Chindelevitch

**Affiliations:** School of Computing Science, Simon Fraser University, Burnaby, BC, Canada; La Jolla Institute for Allergy and Immunology, UNITED STATES

## Abstract

Phylogenetic trees are frequently used in biology to study the relationships between a number of species or organisms. The shape of a phylogenetic tree contains useful information about patterns of speciation and extinction, so powerful tools are needed to investigate the shape of a phylogenetic tree. Tree shape statistics are a common approach to quantifying the shape of a phylogenetic tree by encoding it with a single number. In this article, we propose a new resolution function to evaluate the power of different tree shape statistics to distinguish between dissimilar trees. We show that the new resolution function requires less time and space in comparison with the previously proposed resolution function for tree shape statistics. We also introduce a new class of tree shape statistics, which are linear combinations of two existing statistics that are optimal with respect to a resolution function, and show evidence that the statistics in this class converge to a limiting linear combination as the size of the tree increases. Our implementation is freely available at https://github.com/WGS-TB/TreeShapeStats.

## Introduction

Phylogenetic trees are widely used in biology to represent evolutionary relationships between species. In these trees, the leaves represent extant species while the internal branches indicate hypothesized speciation events [1].

The shape of a phylogenetic tree reveals useful information about its growth process, and can be used to infer the rates of species formation and extinction. Therefore, one of the main applications of phylogenetic trees is to study cladogenesis [2]. Measuring the degree of imbalance or asymmetry of a tree topology can provide support for the hypothesis that species have different potential for speciation [3].

Tree shape statistics are summary measures used to quantify some aspect of the shape of a phylogenetic tree. Several statistics have been proposed for measuring the level of asymmetry of a tree in the literature. These statistics only depend on the topology of the tree, so leaf labels and branch lengths are ignored in their study. It is commonly believed that the evolutionary processes that have produced a phylogenetic tree are reflected in the raw topology of the tree [4]. Tree shape statistics differ in the way they are calculated, and, to some extent, in behaviour [5, 5–17]. Among imbalance-based statistics, two of the most commonly used ones are the Sackin index [5, 12] and the Colless index [13]. The Sackin index is the average path length from a leaf to the root of the tree [3]. The Colless index is the sum of absolute values |*r* − *s*| for all internal nodes, where *r* and *s* are the number of leaves in the left and right subtree of a node, respectively [3]. McKenzie and Steel [18] have proposed the use of the number of cherries, i.e. the number of nodes with two leaf descendants, as a simple tree shape statistic.

Tree shape statistics have been used as tools to test stochastic models of evolution [9]. The equal rate Markov model *(ERM or Yule)* [19, 20] and the the proportional-to-distinguishable arrangements model *(PDA)* [9, 21] are among the most common stochastic models of evolutionary tree growth. The Yule model is a simple model of speciation. At any step, each of the extant species has an equal probability of giving rise to a new species, and at the end, labels are assigned uniformly at random to the leaves. Under this model, different trees with the same number of leaves have different probabilities [22]. Under the *(PDA)* model there is no special model of growing trees, and each possible labeled tree with *n* leaves has the same probability. The frequency of each phylogeny with *n* leaves is proportional to the number of different trees which share this topology [9].

Tree shape statistics have also found applications in phylodynamics (a new field which is at the intersection of phylogenetics and epidemic dynamics of viruses), where recent research shows that phylogenetic tree shapes can help resolve disease transmission patterns. Colijn et.al. [23] showed that the topological structures of phylogenetic trees contain information of the transmission patterns underlying an outbreak. Leventhal et.al. [24] investigated the problem whether the shape of a phylogenetic tree inferred from a pathogen population depends on the contact structure underlying that tree. Another problem in phylodynamics is investigated in [25]; Frost et.al. consider how population structure affects the shape and the structure of a viral phylogeny in the absence of strong selection at the population level. Tree information is also central in some predictive models for short-term influenza evolution and models of fitness [26, 27]

The power of eight tree shape statistics including Sackin and Colless in detecting nonrandom diversification has been evaluated by Agapow *et al* [14]. They simulated phylogenetic trees under two models. In the first model, evolution rates depended on the value of an evolving trait, and in the second model, a lineage’s rate decreased as a function of the time since the last speciation event it experienced. The distributions of these eight statistics under the *ERM* model were calculated and used as a reference to compare with the distribution of these statistics under age-dependent rates and trait-dependent rates. The result shows that the rank ordering of the different measures in terms of power varies with tree size and, more markedly, with the process used to generate imbalance. Indeed, the two scenarios simulated in [14] leave different imbalance signatures, and different measures are more sensitive to imbalance in different parts of the tree. When the rates are based on age, the imbalance is spread fairly evenly between nodes close to the root and far away from the root. When the rates are based on trait values, however, the imbalance is concentrated around the root of the tree.

M. Blum *et al* [3] evaluated the power of the Sackin index, the frequency of subtrees *f*_*n*_(*z*), and a statistic called *D*, based on the frequency of subtrees, in rejecting the ERM model. They used a biased speciation model with a fixed parameter *p*; given a lineage with speciation rate *r* that splits, one of the descendants gets the rate *pr*, and the other one, (1 − *p*)*r*. They simulated this model for a different number of species and different values of *p*. The result shows that *f*_*n*_(*z*) performs poorly, while the Sackin and *D* statistics are very powerful [3]. Matsen [28] and Kirkpatrick and Slatkin [6] have also evaluated the power of some imbalance statistics. Both of these studies concluded that the Sackin and Colless indices were two of the most powerful statistics in distinguishing between tree shapes. Our work builds on ideas from Matsen [28] for evaluating the power of a tree shape statistic.

The *resolution* is the operational definition of performance for tree shape statistics, and it measures the discriminatory power of a tree shape statistic. In this paper we propose a new resolution function based on the Laplacian matrix instead of the distance matrix. Since computing the Laplacian matrix is faster than computing the distance matrix of a graph, the overall time complexity is reduced in comparison with previous methods while producing comparable results. The lower time and space complexity of the new resolution function enables us to easily explore the space of trees with more leaves.

The rest of the article is organized as follows. We begin by introducing the basic notation and facts on phylogenetic trees that will be used throughout the article. We then define our proposed resolution function and our suggested statistic. We continue by presenting the results of our experiments, and lastly, we discuss the results and provide directions for future work.

## Materials and methods

One of the most important challenges in phylogenetics is to find a powerful tool to measure the degree of imbalance of a phylogenetic tree. If all species of a group are equally likely to speciate, then it is unlikely to have a completely asymmetric or a completely symmetric tree. Equal speciation rates will result in a random tree shape which lies between these two extremes. In order to analyze the topology of a phylogenetic tree, different tree shape statistics have been introduced in the literature so far. There is a need to evaluate the discriminatory power of these different statistics in a systematic way. A geometric method for this purpose was introduced by Matsen [28], based on a matrix of pairwise distances between a set of trees with a given size. Here we are proposing a different approach, based on the closely related, but computationally more tractable Laplacian matrix.

In this section we describe the previously proposed resolution function *R*_*D*_(*f*) and our new proposed resolution function *R*_*L*_(*f*), and compare their time and space complexity. We also define a new class of tree shape statistics and compare them to some well-known statistics. We show that these statistics achieve as good or better performance on discriminating trees in comparison with the existing ones. We begin this section with some definitions that we will use through this paper.

### Definitions

Given a phylogenetic tree *T*, a leaf (also called an external node) of *T* is a node of degree one. An internal node of *T* is any non-leaf node of the tree; we represent the set of all internal nodes of a tree by I, and the set of all leaves (or external nodes) by L.

A phylogenetic tree can be rooted or unrooted. A rooted tree is a tree in which a particular internal node called the root is distinguished from the others; it is postulated to be the ancestor of all the other nodes in the tree. In a rooted tree *T*, the parent of a node *i* is the node preceding it on the unique path from the node to the root *r* of *T*; all nodes of *T* except its root *r* have a parent. A child of a node *i* is any node whose parent is *i*.

Given a node *i* of *T*, an ancestor node of *i* is a node on the unique path from *i* to the root of *T*. The descendants of *i* are all the nodes of *T* that have *i* as an ancestor node.

A phylogenetic tree is bifurcating if all its internal nodes have exactly two children. In this paper, we consider rooted bifurcating phylogenetic trees with *l* leaves. It can be easily proven that a rooted bifurcating tree with *l* leaves has exactly (*l* − 1) internal nodes [29]. The number *n* of unlabeled trees on *l* leaves grows exponentially with *l*—asymptotically, *n* ∼ *b*^*l*^*l*^−3/2^, where *b* ≈ 2.483 [28].

The depth of a node *i* is defined as the number of edges on the unique path from the root of *T* to *i*; the root is the only node at depth 0. The height of *i* is defined as the number of edges on the longest path from *i* to a leaf of *T*. The height of a tree is defined as the height of its root.

The subtree of *T* rooted at *i* is the tree induced by *i* and all of its descendants in *T*.

We denote the subtrees of *T* rooted at the left and right children of an internal node *i* by *R*_*i*_ and *S*_*i*_, respectively. Their numbers of leaves are respectively denoted by *r*_*i*_ and *s*_*i*_.

*N*_*i*_ represents the number of internal nodes on the path between node *i* and the root of the tree *r*, and it is equal to the depth of node *i*.

*M*_*i*_ represents the height of the subtree rooted at an internal node *i*.

*I*_*c*_ is the value of the Colless index and $\bar{N}$ is the value of the Sackin index, which are defined below. Roughly speaking, they both measure imbalance, with the Colless index aggregating a measure of local imbalance over the internal nodes and the Sackin index summing the lengths of the root-leaf paths. The more balanced the tree, the lower these values become.

$$\begin{array}{cc} I_{c} & =\frac{2}{\left(n-1)(n-2\right)}\sum_{i\in I}\left|r_{i}-s_{i}\right| \end{array}$$

$$\begin{array}{cc} \bar{N} & =\frac{1}{n}\sum_{j\in L}N_{j} \end{array}$$

We also use the following statistics, also used in [28], in our comparison. Roughly speaking, *I*_2_ measures the imbalance inversely weighted by the total size of the subtree rooted at each internal node. *σ*^2^ is the variance of the lengths of the root-leaf paths. *B*_1_ and *B*_2_ are, once again, locally weighted variants of imbalance metrics.

$$I_{2}=\frac{1}{\left(n-2\right)}\sum_{\begin{array}{c} j\in I\cup \left\{r\right\}\\ r_{j}+s_{j}>2 \end{array}}\frac{|r_{j}-s_{j}|}{|r_{j}+s_{j}-2|}$$

$$\sigma_{n}^{2}=\frac{1}{n}\sum_{i\in L}{\left(\bar{N}-N_{i}\right)}^{2}$$

$$B_{1}=\sum_{j\in I}M_{j}^{-1}$$

$$B_{2}=\sum_{j\in L}\frac{N_{i}}{2^{N_{i}}}$$

**Fig 1:**
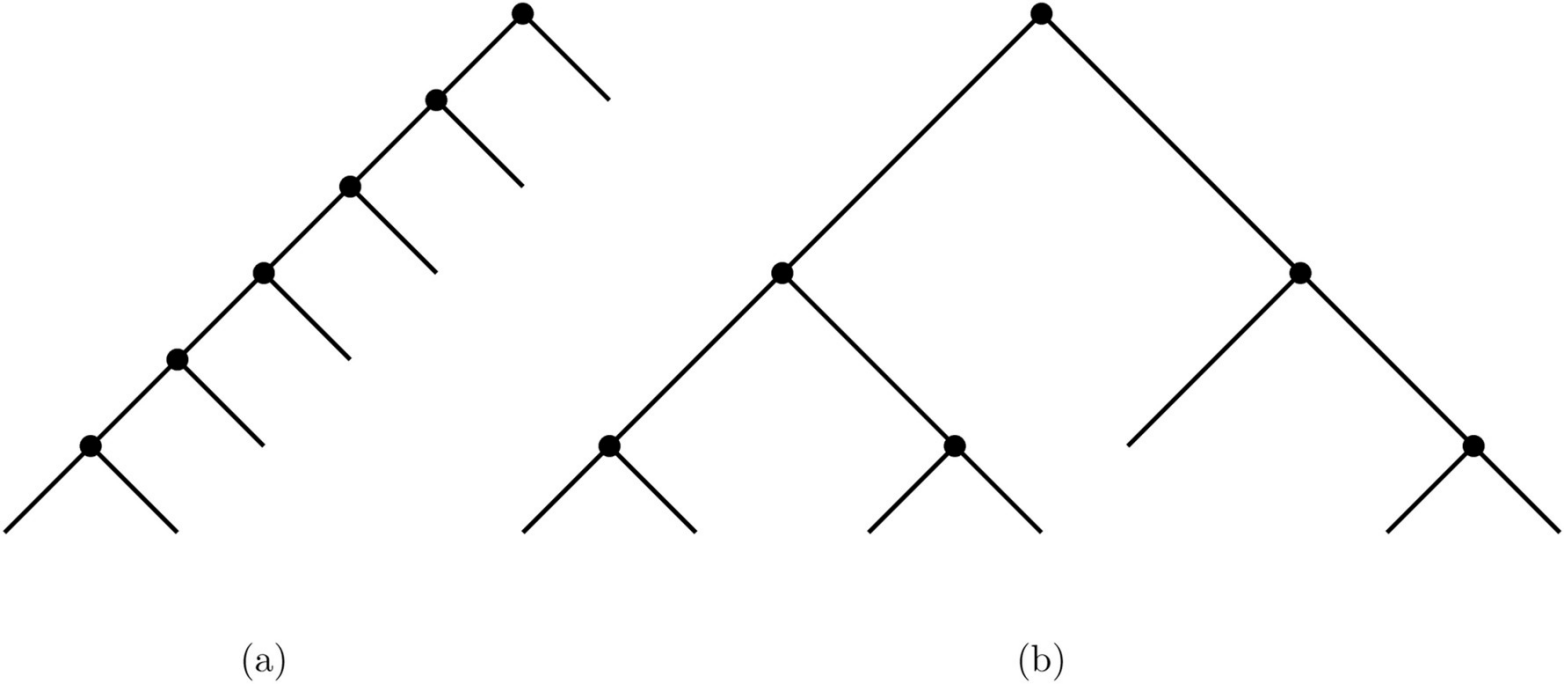
Two different tree shapes with 7 tips. (a) shows the caterpillar (completely asymmetric tree) on 7 leaves, and (b) shows the maximally balanced (completely symmetric tree) tree on 7 leaves.

A rooted caterpillar (or the completely asymmetric tree) is the unique unlabeled binary phylogenetic tree *T* such that all the internal nodes of *T* have a leaf child [22], see Fig 1a.

If *i* is an internal node of *T*, the balance value of *i* is *bal*_*T*_(*i*) = |*r*_*i*_ − *s*_*i*_|, and an internal node of *T* is balanced if *bal*_*T*_(*i*) ≤ 1. A phylogenetic tree is maximally balanced (completely symmetric) if all of its internal node are balanced, and there is a unique maximally balanced phylogenetic tree with *l* leaves, up to isomorphism [22], see Fig 1b.

### Metric

Before going through the details of our suggested resolution function and statistics, we discuss the concept of similarity in the space of trees. We use a metric on unlabeled trees to formalize the notion of similar and different for trees. The aim of this paper is to study the topology of a tree, and identifying the taxa is not a concern of tree shape statistics, so leaf labels and branch lengths are ignored.

A metric *g* is a non-negative and real-valued function on pairs of objects in a collection (called a metric space) *M* such that three constraints are met:

1. Identity: *g*(*x*, *y*) = 0 if and only if *x* = *y*
2. Symmetry: *g*(*x*, *y*) = *g*(*y*, *x*) for all *x*, *y* ∈ *M*
3. Triangle inequality: *g*(*x*, *y*) + *g*(*y*, *z*) ≥ *g*(*x*, *z*) for all *x*, *y*, *z* ∈ *M*

In this paper we use the nearest neighbor interchange (NNI) distance to compare pairs of trees. As we show in the Results section, the NNI metric is an appropriate distance for separating the trees because it produces small changes in each step.

#### Nearest neighbor interchange metric

**Fig 2:**
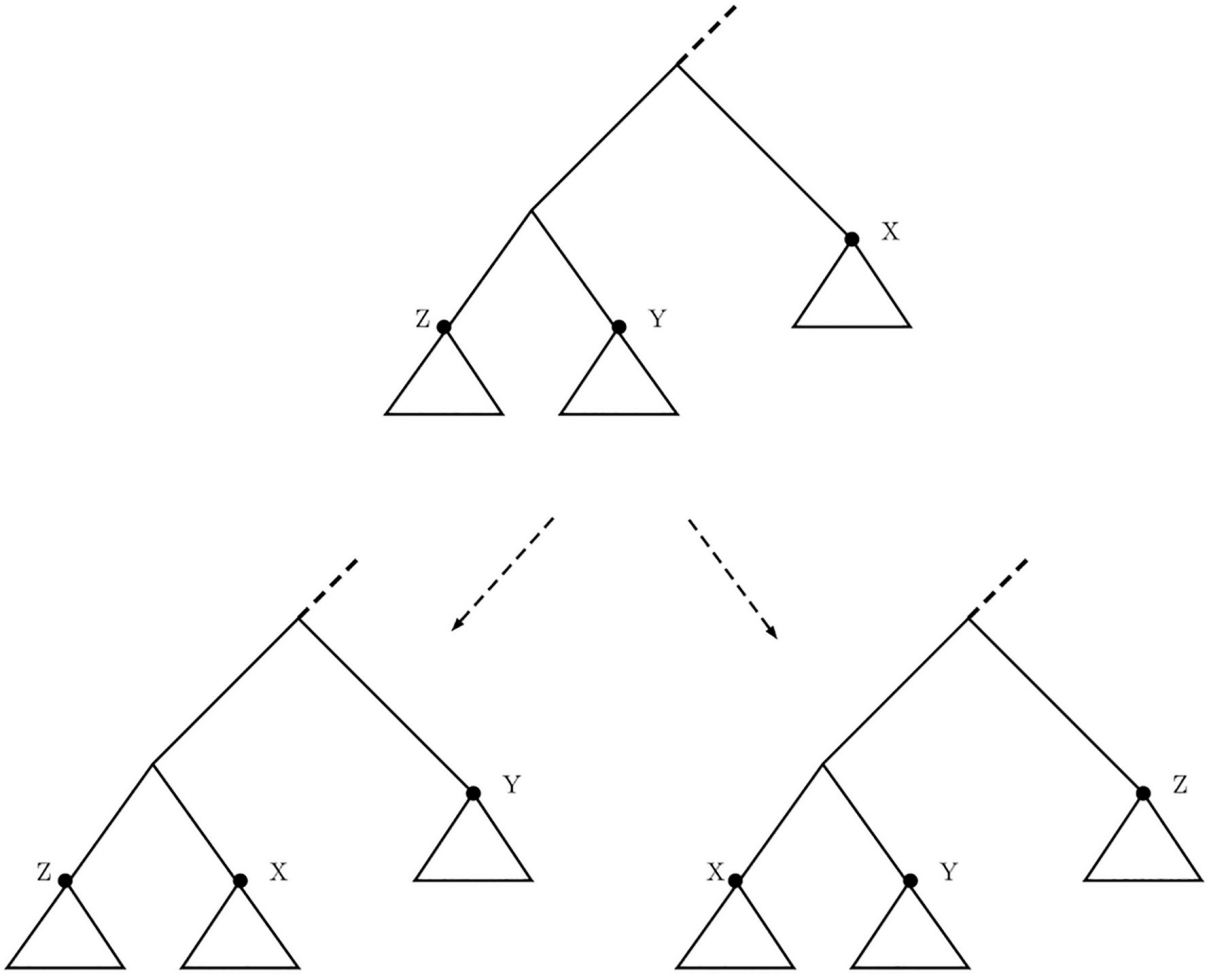
Two possible NNI moves. Two possible configurations for an NNI move on a rooted tree.

A single NNI operation swaps two subtrees that are separated by an internal edge (an edge is internal if neither of its endpoints is a leaf). The two possible NNI moves are depicted in Fig 2, taken from [28]. A phylogenetic tree with *l* leaves has *O*(*l*) neighbors that can be obtained from it via an NNI operation. The unlabeled NNI distance from one tree to another is defined as the minimum number of NNI operations required to transform one tree into the other. [28, 30].

Computing this metric is NP-hard [30], and we have only computed it for the space of trees with at most 17 leaves. To compute the NNI distance between each pair of trees on *l* leaves, we use the *nni* command of the *phangorn* package [31] in the R statistical computing language [32], which produces the list of all trees at NNI distance 1 from a specified tree. We then create the Cayley graph using the *igraph* package [33]. This Cayley graph has a vertex for every tree on *l* leaves, and an edge connecting any two trees at distance 1 (i.e. a single NNI move apart). Finally, we compute the NNI distance between every pair of trees on *l* leaves by using an all-pairs shortest paths algorithm on the Cayley graph [34–38].

### Resolution of statistics

The *resolution* is the operational definition of performance for tree shape statistics, and it measures the discriminatory power of a tree shape statistic. We evaluate the power of previously published statistics by using two different resolution functions, *R*_*D*_ and *R*_*F*_, which we describe in this section.

#### Geometric approach

**Fig 3:**
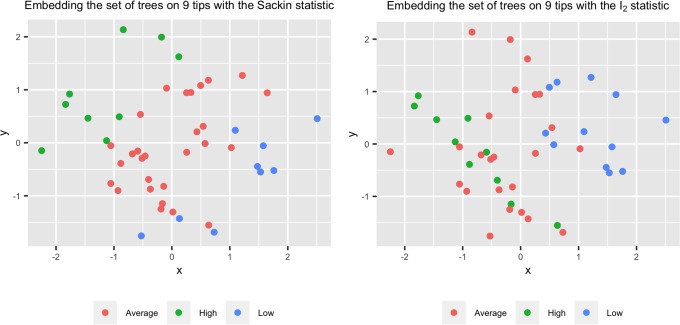
The geometric perspective of a good and bad tree shape statistic. From a geometric perspective, a good statistic can discriminate between different trees, and place similar trees together. In theses two figures, we embedded the set of trees with 9 tips using multi-dimensional scaling *(MDS)* and the *NNI* distance between the trees. The points in the top and bottom plots are colored based on their Sackin and *I*_2_ values respectively. The green, blue and red points correspond to the upper quartile, lower quartile, and the inter-quartile interval of the distribution of the statistics, respectively. The clustering pattern in the top figure indicates that the Sackin index can separate the trees into groups in a way consistent with the NNI distances, while the *I*_2_ index is unable to do so. This can also be seen from the resolution values for these two statistics, described below.

A geometric resolution function has been proposed by Matsen for evaluating different tree shape statistics [28]. This resolution function is based on the intuition that the value of a good statistic should be similar for similar trees, and different for trees with different topology. This intuition is summarized in Fig 3. In this figure, two statistics are used to evaluate the space of trees with 9 tips. We embedded the set of trees with 9 tips on the two dimensional space using a multi-dimensional scaling (MDS) of the nearest neighbor interchange *(NNI)* distances between them. We then colored the points according to the values of the Sackin and *I*_2_ statistics. The clustering pattern induced by the values of the Sackin index in the top figure indicates that the Sackin index can distinguish between dissimilar trees well. On the other hand, the bottom figure indicates that the values of the *I*_2_ statistic are not necessarily different for different trees, and so it induces a random-looking color pattern on the set of trees with 9 tips.

Let *l* denote the number of leaves, let *n* denote the number of possible trees on *l* leaves, let *d*_*ij*_ denote the distance between trees *i* and *j*, and let *H* denote the *n* × *n* “centering matrix”, defined by:

$$\begin{array}{c} H≔I-\frac{1}{n}11^{t}. \end{array}$$

Here, 1 is a vector with every entry equal to one and 1^*t*^ is the transpose of this vector. The application of the centering matrix to a vector results in subtracting the mean from every component of the vector.

Assume that we are given a tree shape statistic *f*, and let *y*_*f*_ be a vector of size *n* whose *i*-th component is the value of the statistic *f* for the *i*-th tree. Assume that *f* is not constant on all the trees so that we can define the centered normalized vector of statistics *x*_*f*_ for the *n* trees as follows:

$$\begin{array}{c} x_{f}≔Hy_{f}/∥Hy_{f}∥ \end{array}$$

The resolution of the statistic *f* with respect to a distance matrix *D* = (*d*_*ij*_) (which can be based on the NNI or the SPR distance) is defined in Eq (1) [28]:

$$\begin{array}{c} R_{D}\left(f\right)≔\frac{1}{2}\sum_{i,j}-{d_{ij}}^{2}{\left(x_{f}\right)}_{i}{\left(x_{f}\right)}_{j}=-\frac{1}{2}x_{f}^{t}D_{s}x_{f} \end{array}$$ (1)

Here *D*_*s*_ represents the component-wise matrix square of *D*, so that the *ij*-th component of *D*_*s*_ is *d_ij_*^2^. The higher the resolution value of a statistic, the more powerful it is from the geometric perspective. The goal is to maximize *R*_*D*_(*f*). It is easy to see that each term −*d*_*ij*_^2^(*x*_*f*_)_*i*_(*x*_*f*_)_*j*_ is maximized when $x_{f_{i}}$ is very negative and $x_{f_{j}}$ is very positive, or vice versa, which means the value of a good statistic is similar for similar trees and different for different trees. This summation is also weighted by the distance, which means that pairs of trees that are a large distance apart contribute more than pairs of trees that are a small distance apart [28].

The geometric resolution is motivated by the statistical method of multidimensional scaling (MDS). The MDS method looks for a set of points *p*_1_, …, *p*_*n*_ in *K*-dimensional Euclidean space that minimize the discrepancy between the true distances and the Euclidean distances:

$$\begin{array}{c} {\left[\sum_{i<j}(d_{ij}-|p_{i}-p_{j}{\left|\right)}^{2}\right]}^{1/2} \end{array}$$

where *d*_*ij*_ is the distance between tree *i* and tree *j* in the given metric. The Euclidean distance between this set of points approximates the distance between the trees. To find the optimal points in *K*-dimensional Euclidean space, the eigenvectors and eigenvalues of $X_{D}=-\frac{1}{2}HD_{s}H$ are used [28].

#### Our proposed resolution

In this paper we propose a new resolution function based on the Laplacian matrix instead of the distance matrix. Since computing the Laplacian matrix is faster than computing the distance matrix of a graph, the overall time complexity is reduced in comparison with previous methods.

The Laplacian matrix (*L*) is a matrix representation of a graph and is defined as follows:

$$L\left(i,j\right)=\{\begin{array}{cc} \deg(v_{i}) & \;\text{if}\;i=j\\ -1 & \;\text{if}\;v_{i}\;\text{is}\;\text{adjacent}\;\text{to}\;v_{j}\\ 0 & \;\text{otherwise} \end{array}$$

For a given statistics vector *f* and the Laplacian matrix *L* of the graph on all trees with edges between trees at a distance of 1, we define our new resolution function in Eq (2):

$$\begin{array}{c} R_{L}\left(f\right)=x_{f}^{t}Lx_{f} \end{array}$$ (2)

Analogously to the previous section, *x*_*f*_ is the centered normalized vector of the given statistic vector *y*_*f*_.

In contrast with the previous resolution function, for which a higher resolution value indicates a better statistic, here a good statistic has a lower resolution value. As follows from the definition of *R*_*L*_(*f*), we consider only pairs of trees which are adjacent when computing it. Since adjacent trees have similar topologies, a good statistic should assign similar values to them, so the value of the resolution for this statistic should be small.

An alternative interpretation of the Laplacian resolution is based on the idea of energy minimization, inspired by the use of the Laplacian for graph embedding [39]. It follows from the definition of *L* that, for any vector *x*,

$$\begin{array}{c} R_{L}\left(x\right)=\frac{x^{t}Lx}{x^{t}x}=\frac{1}{x^{t}x}\sum_{i∼j}{\left(x_{i}-x_{j}\right)}^{2}, \end{array}$$ (3)

where *i* ∼ *j* means that *i* and *j* are neighbors in the graph. If we think of each tree *i* as being located on the real line according to the value *x*_*i*_ of its statistic, and of each pair of neighboring trees as being connected by an elastic spring with unit spring constant, the total energy of this spring is given by the resolution’s numerator.

Noting that *x*^*t*^ *Lx* does not change if *x* is replaced by *x* + *c* for any constant *c*, we can assume that *x* is a vector with mean 0. If *x* is such a vector, we also have *x*^*t*^*x* = *nE*[*x*^2^] = *E*[*x*^2^] − *E*[*x*]^2^ = *nVar*(*x*), where *Var* denotes the variance. Furthermore, in this case, $\sum_{i,j}{\left(x_{i}-x_{j}\right)}^{2}=2\sum_{i}x_{i}^{2}-2\sum_{i}x_{i}\sum_{j}x_{j}=2x^{t}x$. Thus, the Laplacian resolution of a statistic measures, up to a scalar factor, the fraction of the total energy of the statistic (or variance, if the statistic is transformed to have mean 0) that gets allocated to neighboring trees. A statistic that places similar trees nearby will have low energy (and low variance), and hence, a low resolution value.

**Fig 4:**
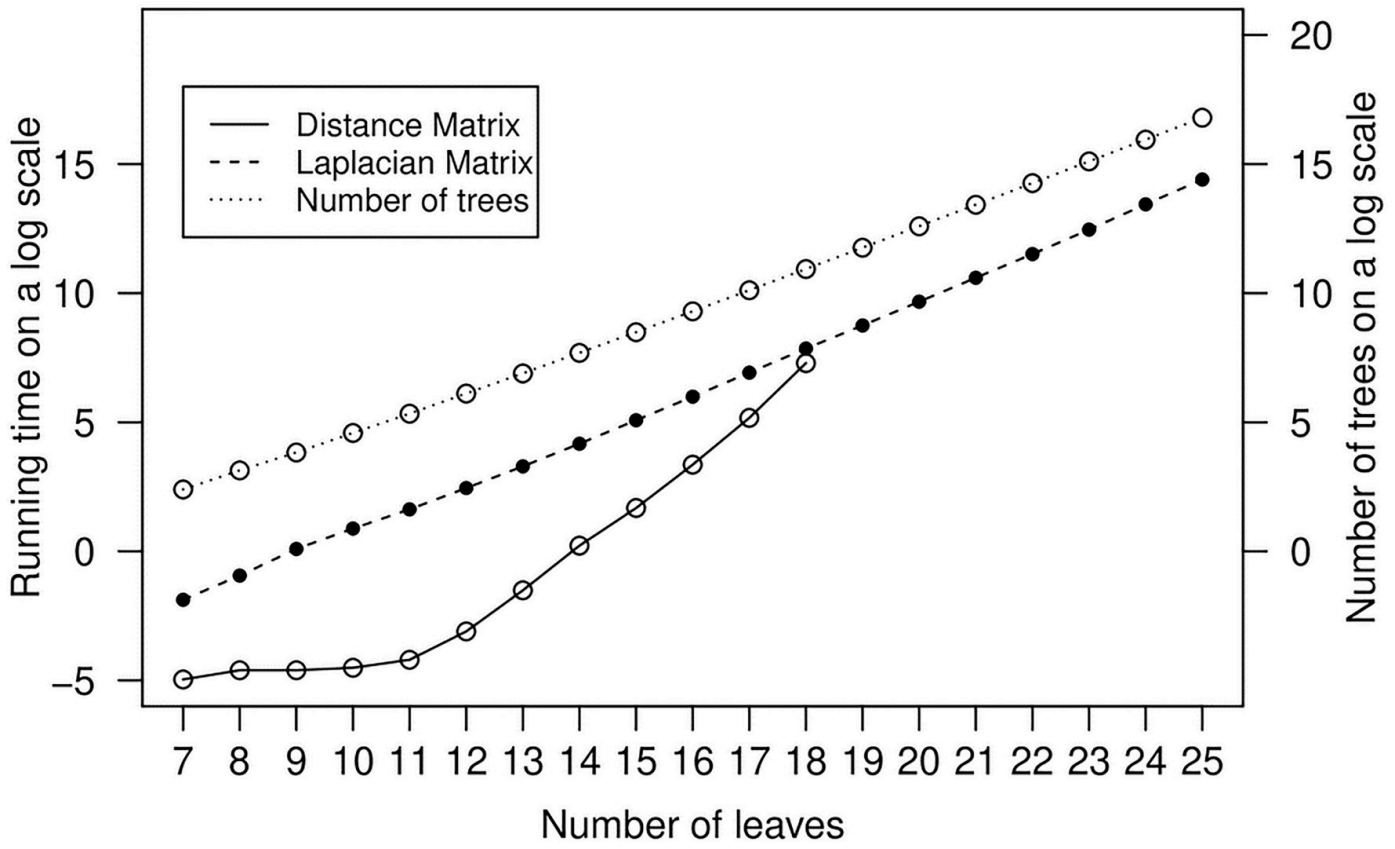
A comparison between the time complexity of the two methods. This plot shows the running time of computing the Laplacian matrix (dashed line) and the distance matrix (solid line) as well as the growth of the number of trees by increasing the number of leaves (dot line). To compute the geometric resolution one must compute the distance matrix; however, to compute our proposed resolution one needs only compute the Laplacian matrix. The left axis shows the time on a log scale. The bottom axis shows the number of leaves, and the right axis shows the number of trees with a specific number of leaves. A comparison between the slopes of the dashed line and the solid line in the plot shows that the running time of our proposed resolution is much faster that that of the previous method.

The superiority of our resolution over the previous one is the lower time and space complexity of its evaluation, so we can easily explore the space of trees up to 25 leaves; this could only be done for the space of trees up to 17 leaves with the previous method [28]. It takes *O*(*n* log *n*) time and space to compute the Laplacian matrix of the Cayley graph of the set of *n* trees with *l* leaves for the NNI distance (since *l* ∈ *O*(log *n*) and the number of non-zero entries in each row of the Laplacian matrix is the degree of the corresponding vertex, which is the number of trees that are within distance 1 from the given tree). On the other hand, computing the distance matrix for the same set of trees takes *O*(*n*^2^) time and space. Given the exponential growth in the set of trees with a fixed number of leaves [19], we are able to go further by decreasing the running time and space complexity (Fig 4).

#### The upper and lower bounds

We now need to transform the resolution function in order to ensure it is always in the interval [0, 1], for comparison purposes. Following Matsen’s original work [28], we use the Rayleigh quotient to compute the extreme values of the resolution. For a given symmetric matrix *M* and nonzero vector *x*, the Rayleigh quotient *R*(*M*, *x*) is defined as:

$$\begin{array}{c} R\left(M,x\right)=\frac{x^{t}Mx}{x^{t}x} \end{array}$$ (4)

It follows from the Courant-Fischer theorem [40] that the minimum and the maximum values of Rayleigh quotient are equal to the smallest and largest eigenvalues of *M*, respectively.

It follows that *R*_*D*_(*f*) has an upper bound and lower bound which are the maximum and minimum eigenvalues of *X*_*D*_, defined as:

$$\begin{array}{c} X_{D}≔-\frac{1}{2}HD_{s}H \end{array}$$ (5)

The upper bound for *R*_*L*_(*f*) is equal to the largest eigenvalue of *L*. We note that the smallest eigenvalue of *L* is zero and occurs only for constant vectors *x* = *x*_1_1 since the Cayley graph is a connected graph, according to Eq 3; furthermore, by the symmetry of *L*, any other eigenvectors are orthogonal to the constant vector 1. Therefore, the lower bound is equal to the second smallest eigenvalue of *L*, also known as the Fiedler value of the Cayley graph [41].

Having determined the extreme values min and max for the resolution function, we transform the value of the resolution for all statistics to the [0, 1] interval by using the linear transformation $x\to \frac{x-\min}{\max-\min}$; the resulting value is referred to as the scaled resolution.

### The proposed tree shape statistic

In this section we propose a new tree shape statistic which is a meta-statistic obtained by finding the linear combination of existing statistics that results in the optimal resolution.

Here we focus on the linear combination of the Sackin and Colless indices, which we call the Saless index: $Saless=\lambda \bar{N}+I_{c}$.

We choose the value of λ to maximize the resolution, and is different for trees with different numbers of leaves. Our experiments suggest (though we have not formally proven it) that λ will converge to a limiting value as the number of leaves goes to infinity.

In order to find the optimal value of λ we maximize:

$$\begin{array}{c} R_{D}\left(Saless\right)=\frac{{\left(\lambda \bar{N}+I_{c}\right)}^{t}D_{s}\left(\lambda \bar{N}+I_{c}\right)}{{\left(\lambda \bar{N}+I_{c}\right)}^{t}\left(\lambda \bar{N}+I_{c}\right)} \end{array}$$ (6)

If we call the numerator of the resolution *f* and the denominator *g*, the problem reduces to finding λ for which $\frac{f\prime }{g}=\frac{g\prime }{f}$. This condition simplifies to a quadratic equation, and by solving that equation we find the value of λ for trees with up to 17 leaves. In the S1 Appendix, we show that the optimal value of λ is always real; it can sometimes be negative, though in the case of the *Saless* statistic it always appears to be positive. These values are shown in Table 1.

**Table 1:** The value of λ and the resulting resolution *R*_*D*_ for trees with different number of leaves.

*l*	7	8	9	10	11	12	13	14	15	16	17
λ	5.77	0.11	2.38	0.92	1.07	1.21	1.43	1.26	1.3	1.27	1.32
*R*_*D*_	0.931	0.926	0.923	0.943	0.955	0.956	0.957	0.957	0.957	0.956	0.956

**Fig 5:**
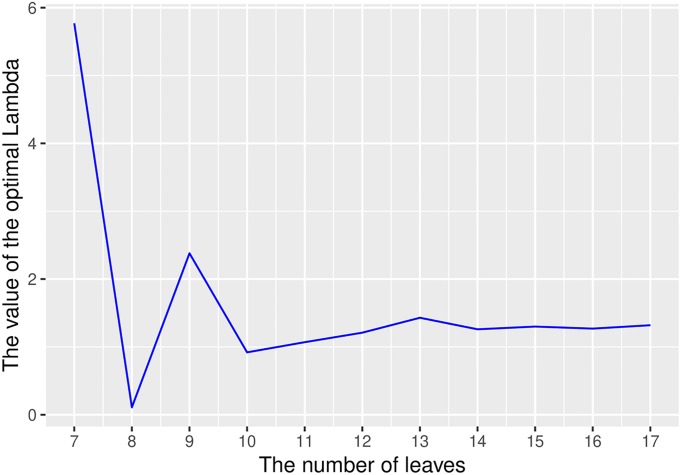
λ converges as the number of leaves grows. The value of λ appears to converge as the number of leaves grows.

Table 1 and Fig 5 suggest that the value of λ may converge to a limit as the number of leaves goes to infinity. However, we were unable to verify the plausibility of this behavior by going beyond *l* = 17, as the number of trees, which is the size of the dense distance matrix *D*_*s*_, grows exponentially with the number of leaves.

In another experiment we evaluated the combination of different pairs of statistics based on our new resolution function. We can show that the optimal coefficient λ gives the linear combination of the two statistics a better resolution than each individual statistic. This linear combination does not always result in a plausible statistic, since the optimal λ is negative for some combinations. We also note that linear combination of statistics perform differently with different resolution functions. The results of these experiments are discussed in detail below.

## Results

In this section we conduct different experiments to analyze our newly introduced statistics and resolution function. First, we use the previously defined resolution function and the NNI metric to compare our suggested statistic with the classical ones, and show that the this new statistic exhibit a better performance. We then use our new proposed resolution function to evaluate different tree shape statistics, showing that this new resolution function can classify good and bad statistics. The advantage of this resolution over the previously used one is that its computation reduces the time and space complexity. Lastly, we discuss the optimal pairwise combinations of several other statistics. The following subsections explain each experiment in more detail.

### Comparing the power of our suggested statistics with the classical statistics

In this part, we experiment with the statistics introduced in preliminaries section and our suggested statistic *Saless*.

Table 2 represents the scaled resolution scores for comparison of the various statistics. Each row in the table contains the resolution for trees with a given number of leaves, while each column contains the resolution for each statistic.

**Table 2:** Scaled resolution scores for tree statistics on the NNI distance matrix. The resolution is is between 0 and 1. *l* is the number of leaves. The tree shape statistics are described in the Preliminaries. The highlighted values correspond to the best statistics in each row.

*l*	*I*_*c*_	$\bar{N}$	$\sigma_{n}^{2}$	*I*_2_	*B*_1_	*B*_2_	Saless
7	0.925	0.93	0.902	0.884	0.865	0.925	0.931
8	0.926	0.912	0.875	0.861	0.833	0.911	0.926
9	0.918	0.921	0.883	0.854	0.832	0.907	0.923
10	0.941	0.938	0.898	0.855	0.833	0.908	0.943
11	0.953	0.951	0.91	0.855	0.837	0.913	0.955
12	0.953	0.952	0.909	0.85	0.831	0.904	0.956
13	0.954	0.954	0.908	0.842	0.825	0.899	0.957
14	0.955	0.955	0.907	0.837	0.82	0.89	0.957
15	0.955	0.954	0.905	0.83	0.813	0.883	0.956
16	0.954	0.954	0.903	0.827	0.809	0.874	0.956
17	0.953	0.953	0.901	0.82	0.802	0.868	0.956

As this table shows, our proposed statistic has higher resolution than the previously defined ones.

### New resolution function

We introduced a new resolution function based on the Laplacian matrix to evaluate different tree shape statistics. Table 3 shows that we can get results for the space of trees up to 25 leaves.

**Table 3:** Scaled resolution scores for the classical tree shape statistics based on our resolution function. *l* is the number of leaves. The best classical statistic is *Colless* and the worst ones are *B*_1_ and *I*_2_ (the same ranking as for the previous resolution function). The highlighted values correspond to the best statistics in each row.

l	*I*_*c*_	$\bar{N}$	$\sigma_{n}^{2}$	*I*_2_	*B*_1_	*B*_2_
7	0.0984	0.0933	0.1082	0.1115	0.1179	0.0989
8	0.0808	0.0955	0.1110	0.0893	0.1164	0.0965
9	0.0507	0.0566	0.0662	0.0680	0.0797	0.0653
10	0.0327	0.0379	0.0471	0.0535	0.0629	0.0451
11	0.0222	0.0255	0.0326	0.0458	0.0511	0.0348
12	0.0183	0.0217	0.0282	0.0429	0.0473	0.0304
13	0.0160	0.0185	0.0238	0.0413	0.0441	0.0283
14	0.0147	0.0170	0.0217	0.0400	0.0421	0.0265
15	0.0137	0.0157	0.0197	0.0390	0.0404	0.0256
16	0.0130	0.0148	0.0184	0.0380	0.0389	0.0246
17	0.0123	0.0140	0.0170	0.0370	0.0375	0.0238
18	0.0117	0.0132	0.0160	0.0358	0.0361	0.0229
19	0.0112	0.0126	0.0150	0.0349	0.0349	0.0222
20	0.0107	0.0120	0.0141	0.0339	0.0338	0.0216
21	0.0102	0.0114	0.0133	0.0329	0.0327	0.0209
22	0.0098	0.0109	0.0126	0.0319	0.0316	0.0203
23	0.0094	0.0105	0.0120	0.0311	0.0306	0.0197
24	0.0090	0.0100	0.0114	0.0302	0.0297	0.0192
25	0.0086	0.0096	0.0108	0.0294	0.0288	0.0186

### Linear combination of the classical tree shape statistics

We investigate pairwise linear combinations of statistics based on our new resolution function. The linear combination of Colless and *B*_2_ performs better than all other statistics, but the value of the optimal λ is negative for the space of trees up to 14 leaves. Similarly, the linear combination of Colless and Sackin results in a high resolution, but is not a plausible statistic as the optimal values of λ are negative. The results of this experiment are summarized in Table 4.

**Table 4:** Scaled resolution scores for the optimal linear combinations of *I*_*c*_ − *B*_2_, *B*_2_-*B*_1_, and *Saless* based on our new proposed resolution function. The corresponding optimal values of λ are shown next to each combination.

*l*	*I*_*c*_ − *B*_2_	λ	*B*_2_-*B*_1_	λ	Saless	λ
7	0.0922	-0.08	0.0855	2.89	0.0932	0.08
8	0.0799	-0.28	0.0884	3.43	0.076	-1.2
9	0.0505	-0.53	0.0576	3.2	0.0502	-2.36
10	0.0324	-0.3	0.0405	4.14	0.0323	-2.49
11	0.0221	-0.5	0.0306	4.22	0.0221	-4.3
12	0.0182	-0.49	0.0273	4.91	0.0181	-2.87
13	0.0160	-1.4	0.0256	5.14	0.0159	-3.56
14	0.0147	-3.58	0.0244	5.69	0.0146	-3.19
15	0.0137	1.31	0.0237	6.03	0.0136	-3.17
16	0.0129	0.67	0.0230	6.5	0.0128	-2.8
17	0.0123	0.41	0.0224	6.86	0.0122	-2.69
18	0.0116	0.3	0.0217	7.28	0.0115	-2.52
19	0.0111	0.24	0.0212	7.65	0.0110	-2.4
20	0.0105	0.2	0.0206	8.04	0.0105	-2.27
21	0.0100	0.17	0.0200	8.4	0.0100	-2.17
22	0.0096	0.14	0.0195	8.77	0.0096	-2.08
23	0.0092	0.13	0.0190	9.12	0.0092	-2.00
24	0.0088	0.11	0.0185	9.47	0.0088	-1.94
25	0.0084	0.10	0.0180	9.82	0.0084	-1.88

## Discussion and future work

In this paper, we proposed a new resolution function based on the Laplacian matrix to evaluate different tree shape statistics. As we show in the Results section, this resolution function can rank the statistics in terms of their power in discriminating all possible phylogenetic trees on the same number of leaves. Among our new resolution function and the previously proposed ones, the top statistics are *Colless*, *Sackin*, and our proposed statistic (their linear combination), and the worst ones are *B*_1_ and *I*_2_. The advantage of our new resolution function is to reduce the time and space complexity of the computation while producing comparable results. This allows us to ensure that the trends observed with smaller trees are not artifactual, and remain when we explore larger trees.

We have implemented our proposed method in the *R* statistical computing language [32]. The challenge of implementing the method was in handling large matrices, as the number of unlabeled trees *n* grows exponentially with the number of leaves *l*. Our implementation needs to allocate a vector of size *n*^2^, which is not possible since *R* holds all objects in virtual memory and each object can use a limited amount of memory. One of the advantages of using the Laplacian matrix in our method is its sparsity, which enables us to implement it via the *Matrix* package. We also use a specific numbering scheme for labeling the phylogenetic trees to account for tree isomorphism, which results in reduced time and memory requirements. A better implementation would allow us to extend distance and Laplacian matrix computations to larger tree sizes. An alternative approach, suggested by one of the anonymous reviewers, is to reach larger tree sizes by replacing the exact computation that we pursue here with a Monte Carlo Markov Chain approach, which is feasible because the neighbours of each tree with respect to a rearrangement distance can be readily produced.

In the Methods section we investigated the optimal combination of different pairs of tree shape statistics. We conjecture that λ values converge for any pair of reasonable statistics. We cannot make any conclusion based on the small trees we examined so far, since convergence is a long-term behavior, and we leave the proof of this conjecture for future work. Regarding the application of tree shape statistics to phylodynamics, more powerful statistics, such as the pairwise combinations we introduced, are clearly needed. Tree shape statistics are used, for instance, as the features in predictive models of short-term influenza evolution and fitness models [26, 27]. Using more highly resolving features would arguably result in more accurate predictions. An additional future research direction could then be the extension of optimal combinations to more than 2 statistics, in which case one would need to optimize multiple coefficients at once.

## Supporting information

S1 Appendix (PDF)
