# Supplementary material for: A new resolution function to evaluate tree shape statistics
Source: PLoS One. 2019 Nov 21;14(11):e0224197. doi: 10.1371/journal.pone.0224197 (PMC6874070; doi:10.1371/journal.pone.0224197)
Supplement: S1 Appendix — (PDF) [file pone.0224197.s001.pdf]

S1 Appendix: Supporting information

Lemma. *The optimal λ value for the combination of a pair of statistics is always a real number.*

Proof. Consider linear combinations of the form $\lambda S_1 + S_2$, where S_1 and S_2 are vectors corresponding to two distinct real statistics. Without loss of generality we make two assumptions:

- S_1 and S_2 are orthogonal; this is because we can always write $S_2 = \alpha S_1 + S_3$, with $\alpha \in \mathbb{R}$ and S_3 orthogonal to S_1, and the resolution of $\lambda S_1 + S_2$ equals the resolution of $(\lambda + \alpha)S_1 + S_3$, in which the coefficient $\lambda + \alpha$ of S_1 is real if and only if λ is real, as $\alpha \in \mathbb{R}$.
- D_s is a real symmetric matrix (true for both resolutions).

Under these two assumptions, the aim is to find the value of λ that maximizes the resolution of $\lambda S_1 + S_2$ when S_1 and S_2 are orthogonal:

$$R_D(\lambda S_1 + S_2) = \frac{(\lambda S_1 + S_2)^t D_s (\lambda S_1 + S_2)}{(\lambda S_1 + S_2)^t (\lambda S_1 + S_2)} \tag{A}$$

Let us call the numerator and denominator of this equation f and g, respectively. The problem reduces to finding a λ such that the derivative of $\frac{f}{g}$ equals 0, which is equivalent to $f'g = g'f$ by the quotient rule. We thus have:

$$2[S_1^t D_s(\lambda S_1 + S_2)][(\lambda S_1 + S_2)^t(\lambda S_1 + S_2)] = 2[S_1^t(\lambda S_1 + S_2)](\lambda S_1 + S_2)^t D_s(\lambda S_1 + S_2)$$

After some algebra, the coefficient of λ^3 cancels out and we end up with the quadratic equation:

$$(S_1^t D_s S_2 S_1^t S_1)\lambda^2 + (S_2^t D_s S_2 S_1^t S_1 - S_1^t D_s S_1 S_2^t S_2)\lambda - (S_1^t D_s S_2 S_2^t S_2) := a\lambda^2 + b\lambda + c = 0$$

The discriminant $b^2 - 4ac$ of the quadratic function determines whether its roots are real. In this case, we note that $S_1^t S_1$ and $S_2^t S_2$ are non-negative real numbers, and we can easily see that the discriminant of the above equation is always non-negative, since the term $-4ac$ above is a perfect square and thus non-negative. Therefore, its roots are real, and so is λ. □
