# Supplementary material for: A new resolution function to evaluate tree shape statistics
Source: PLoS One. 2019 Nov 21;14(11):e0224197. doi: 10.1371/journal.pone.0224197 (PMC6874070; doi:10.1371/journal.pone.0224197)
Supplement: Attachment — Submitted filename: _Addressing Plos CB reviews.pdf [file pone.0224197.s002.pdf]

Submitted as part of the GLBIO 2019 Collection - FAO: PLOS Collections collections@plos.org

Dear editors of PLOS ONE,

It is our pleasure to submit a revised version of our manuscript, “A new resolution function to evaluate tree shape statistics” for publication in the Special Issue containing the proceedings of the 2019 GLBIO (Great Lakes Bioinformatics) conference.

We appreciate all the reviewers’ comments, which we have addressed, as described in detail below.

Thank you for your consideration.

Sincerely,

Maryam Hayati,
Bita Shadgar,
Leonid Chindelevitch

----------------------- REVIEW 1 ---------------------
TITLE: A new resolution function to evaluate tree shape statistics
AUTHORS: Maryam Hayati, Bita Shadgar and Leonid Chindelevitch

----------- Overall evaluation -----------
My two main comments regarding the initial version of the manuscript were:
1. what can be gained practically from the improvement from 17 to 25 leaf-trees
2. what the small improvements due to the linear combination of statistics can lead to

The authors respond that the "“strong law of small numbers” that states that trends observed at small sample sizes may be unreliable or even spurious" and in the manuscript write: "This allows us to ensure that the trends observed with smaller trees are not artifactual, and remain when we explore larger trees."

Maybe I am not getting is here, but is there really any reason to think the trends from 17-leaf trees is artifactual? If this is really the motivation for research, at least the authors could provide some plausible scenario where the results would be artifactual on 17 leaf trees and not on 25. Maybe I don't "get" the "strong law of small numbers" but it stretches my credulity. Perhaps the authors can try a different line of reasoning to make a more convincing case for the improvement.

1. We thank the reviewer for the comments. As an example of this phenomenon, please consider two tree shape statistics I2 and B1, as shown in Table 3. For the set of trees up to 18 tips, the values of resolution for I2 is smaller than B1. However this trend is reversed for the set of trees with more than 18 tips (see Table 3, columns I2 and B1). In addition to this, the ability to consider larger trees helps to show that the optimal coefficient for the combination of tree shape statistics converges as the size increases.

With regards to my other question, about what impact the improvement due to the linear combination can lead to, the authors do not seem to have addressed this at all in the manuscript. At least some discussion of how the improved statistical power would make a difference for application in phylodynamics (or some other application) would make the impact of the results much more clear to a non-expert reader.

2. On the one hand, the linear combinations of statistics provide a new paradigm for combining pairs (or, indeed, sets) of statistics in a principled way. On the other hand, we have now added the following statements to the Discussion section, at the end of the first paragraph: “Regarding the application of tree shape statistics to phylodynamics, more powerful statistics, such as the pairwise combinations we introduced, are clearly needed. Tree shape statistics are used, for instance, as the features in predictive models of short-term influenza evolution and fitness models [26,27]. Using more highly resolving features would arguably result in more accurate predictions.”

---------------------- REVIEW 2 ---------------------
PAPER: 26
TITLE: A new resolution function to evaluate tree shape statistics
AUTHORS: Maryam Hayati, Bita Shadgar and Leonid Chindelevitch

----------- Overall evaluation -----------
The contribution of this paper is twofold. First, the authors introduce a new resolution function for assessing tree shape statistics, which is asymptotically faster than the current resolution function. Second, the authors show how to optimize linear combinations of pairs of current tree statistics using the current and proposed tree statistics. This is good.

I reviewed the conference version of this paper. My comments have been sufficiently addressed, the revised introduction does a better job of the motivating the problem, and the methods section is easier to read.

Some minor comments:

* Author summary: Phylodynamics => phylodynamics
* Last paragraph: Missing section labels. (Maybe just omit this paragraph.)
* Material and methods (page 3): this statistic => these statistics

Thank you for your precise comments. We have now fixed all the typos in the revised version of the paper.

----------------------- REVIEW 3 ---------------------
PAPER: 26
TITLE: A new resolution function to evaluate tree shape statistics
AUTHORS: Maryam Hayati, Bita Shadgar and Leonid Chindelevitch

----------- Overall evaluation -----------

Since the manuscript has not changed significantly from GLBIO, I will not write a new review. My previous comments were taken into account.

Thank you for your time reviewing the original and the new version, and verifying that we have indeed taken your comments into account.
